# Correlation between spike statistics and T-type calcium channel activation in simulated subthalamic nucleus neurons

**DOI:** 10.1186/1471-2202-15-S1-P125

**Published:** 2014-07-21

**Authors:** Katsunori Kitano

**Affiliations:** 1 Department of Human and Computer Intelligence, Ritsumeikan University, Shiga 5258577, Japan

## 

Firing patterns of subthalamic nucleus (STN) neurons are closely related to behavioral functions such as motor controls. In the patients of the Parkinson disease, which is a representative of basal ganglia disorders, STN neurons show a distinctive firing pattern, rebound bursts. To understand the relation between the STN firing patterns and their functional roles, it is useful to characterize the firing patterns, especially the burst firings. Although several methods have been proposed so far, an objective definition of burst firings is still unclear. On the other hand, the mechanism for the rebound bursts is attributed to the low-voltage-activated calcium channels, also known as T-type calcium channels [1]. The channels are activated by strong inhibitory synaptic inputs from globus pallidal neurons that have reciprocal connections with STN. If we could observed directly T-type calcium currents, the observation could be a criterion to determine whether the firing activity is a burst firing or not.

Here we investigated to what degree spike statistics correlate with an activation level of the T-type calcium currents by simulating an STN neuron model [2]. We employed the following spike statistics of interspike intervals (ISIs) of STN neural activities: (i) coefficient of variation (Cv), (ii) time-local variation (Lv) [3], (iii) chi-squared test statistic between a spike count histogram and a Poisson distribution [4], and (iv)-(vi) Kolmogorov-Smirnov (K-S) statistics in K-S test between an observed and hypothesized ISI distributions (Gaussian, exponential, and gamma distributions).

By comparing multiple correlation coefficients between these statistics and temporally-averaged conductances of the T-type calcium channels under different conditions including burst-preferred parameter sets, we found that the K-S statistic between the observed ISI distribution and the hypothesized exponential one (v) showed the highest correlation with the averaged T-type calcium currents among them under any condition. This would provide a more objective criterion, which is linked to the generation mechanism, to define rebound bursts observed in pathological STN.

## References

[B1] Bevan MD Magill PJ Hallworth NE Bolam JP Wilson CJ Regulation of the timing and pattern of action potential generation in rat subthalamic neurons J Neurophysiol 2002 15 1348 1362 11877509 10.1152/jn.00582.2001

[B2] Otsuka T Abe T Tsukagawa T Song WJ Conductance-based model of the voltage-depedent generation of a plateau potential in subthalamic neurons J Neurophysiol 2004 15 255 264 10.1152/jn.00508.2003 15212440

[B3] Shinomoto S Miyazaki Y Tamura H Fujita I Regional and laminar differences in in vivo firing patterns of primate cortical neurons J Neurophysiol 15 567 575 15758054 10.1152/jn.00896.2004

[B4] Kaneoke Y Vitek JL Burst and oscillation as disparate neuronal properties J Neurosci Methods 1996 15 211 223 10.1016/0165-0270(96)00081-7 8912194

